# Direct Anastomosis of Persistent Left Superior Vena Cava to Right Superior Vena Cava in a Pediatric Patient with Tetralogy of Fallot: an Alternative Technique

**DOI:** 10.21470/1678-9741-2021-0096

**Published:** 2023

**Authors:** Mustafa Yilmaz, Atakan Atalay

**Affiliations:** 1 Department of Congenital Heart Surgery, Ankara State Hospital, Ankara, Turkey.

**Keywords:** Congenital Heart Defects, Hemodynamics, Superior Vena Cava, Left Atrium, Surgical Anastomosis, Cardiac Surgical Procedures

## Abstract

The presence of persistent left superior vena cava to the left atrium connection without an innominate vein may give rise to technical challenges during intracardiac repair. In this report, the end-to-side anastomosis technique of the persistent left superior vena cava to the right superior vena cava is discussed in a patient with tetralogy of Fallot associated with persistent left superior vena cava draining directly into the left atrium. A successful end-to-side anastomosis between the persistent left superior vena cava and the right superior vena cava was performed and short-term anastomosis patency was documented via angiography.

## INTRODUCTION

Persistent left superior vena cava (PLSVC) is the most common congenital thoracic venous system abnormality (0.3-0.5%). In congenital heart patients, the incidence increases approximately ten times and reaches 4.5%. PLSVC is observed in 20% of the patients with tetralogy of Fallot. This association can often be determined by preoperative examinations. However, occasionally, preoperative tests may be insufficient in the diagnosis of this association. The presence of PLSVC to the left atrium connection without an innominate vein may give rise to technical challenges during intracardiac repair when not determined prior to surgical intervention. In such situations, the surgeon should be aware of intra or extracardiac re-routing procedures to facilitate the intracardiac repair and eliminate the right-to-left shunt. In this case report, we will discuss the pros and cons of the end-to-side anastomosis technique of PLSVC to the right superior vena cava (SVC), which is an alternative extracardiac re-routing procedure, in a pediatric patient with tetralogy of Fallot who was admitted to the emergency department with a cyanotic spell attack.

### Case Presentation

A 9-year-old girl was admitted to the emergency department with a deteriorated medical condition, agitation, and deep cyanosis. The patient’s oxygen saturation was 40%, and the diagnosis was confirmed as tetralogy of Fallot with severe infundibular and valvular stenosis via transthoracic echocardiography. Due to the patient’s advanced age, emergency cardiac catheterization was performed to evaluate additional abnormalities. No additional abnormalities were detected via cardiac catheterization.

The patient was urgently operated on to complete repair of the tetralogy of Fallot after obtaining written informed consent.

During the operation, PLSVC was detected, and there was no left innominate vein between the right and left SVC. The diameter of the PLSVC was equal to that of the right SVC, and it was coursing posterior to the left atrial appendage and in front of the left pulmonary artery. Considering the most common anatomy, we anticipated that the PLSVC would drain into the coronary sinus and planned to aspirate it through the coronary sinus during the operation.

Right atriotomy was performed after cardiopulmonary bypass and cardiac arrest. The coronary sinus was visualized in its normal localization and was of normal diameter. The right atrial septum was then incised, and the left atrium was inspected. The PLSVC was temporarily occluded. Inspection of the left atrium revealed that the roof of the coronary sinus was intact, and that the PLSVC was connected to the left atrium directly. Since the diameter of the PLSVC was about the same size as the right SVC, and the measured proximal pressure was 40 mmHg during temporary occlusion, a re-routing procedure for PLSVC became mandatory.

**Fig. 1:**
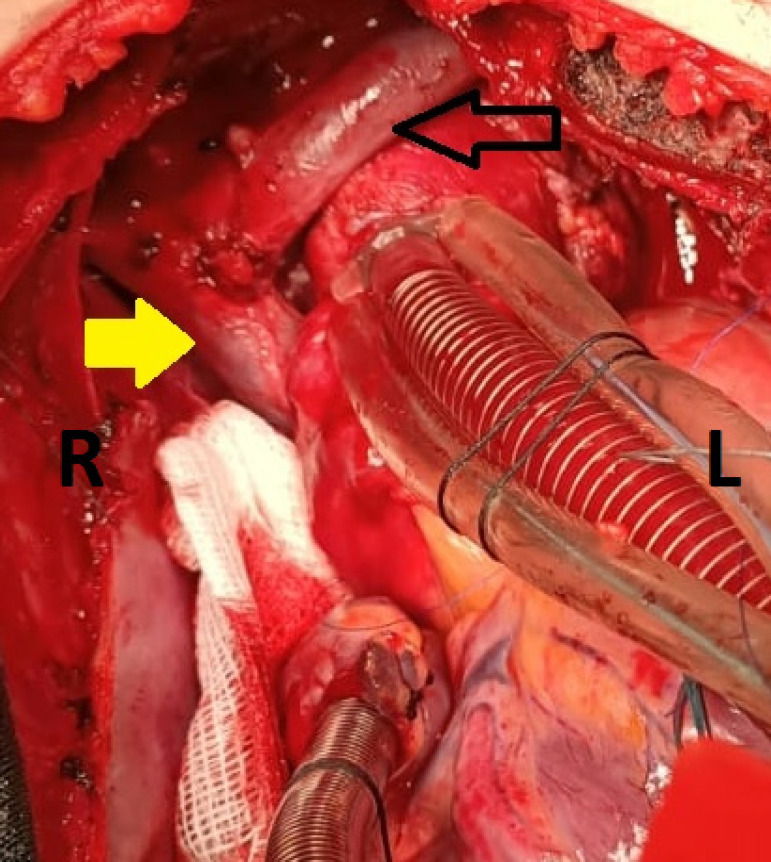
Persistent left superior vena cava was carried over the ascending aorta and anastomosis was performed to the medial aspect of right superior vena cava. Black arrow=persistent left superior vena cava; yellow arrow=right superior vena cava; L=left; R=right

Due to the concern that the ischemic time would increase, we decided to divert the PLSVC drainage to the systemic venous system via an extracardiac route. Complete repair of the tetralogy of Fallot was performed with a transannular patch. Following cross-clamp removal, the PLSVC was transected from the left atrium, and all its proximal branches were ligated and divided under cardiopulmonary bypass support. Then the proximal part of the right SVC was dissected entirely free. The PLSVC was redirected to the right side over the anterosuperior aspect of the ascending aorta, and end-to-side anastomosis to the right SVC was performed. (Figure 1). No kinking or tension was observed on the suture line of the anastomosis.

**Fig. 2:**
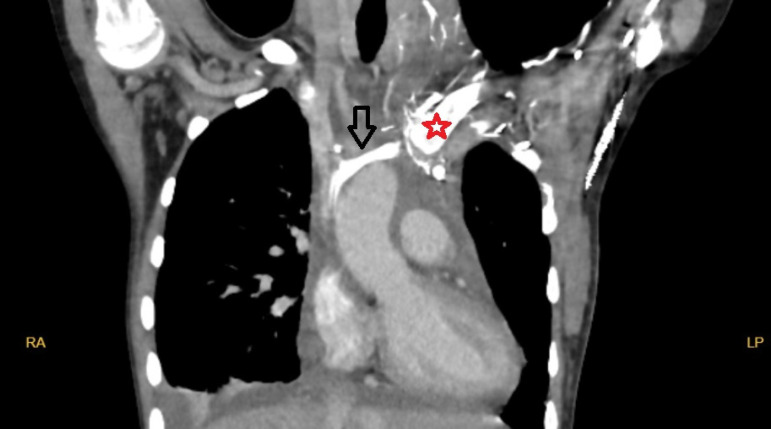
Computed tomography angiographic image of the persistent left superior vena cava. Black arrow=patent persistent left superior vena cava and anastomosis; star=proximal part of clavicula (patient has pectus carinatum deformity); LP=left posterior; RA=right anterior

**Fig. 3:**
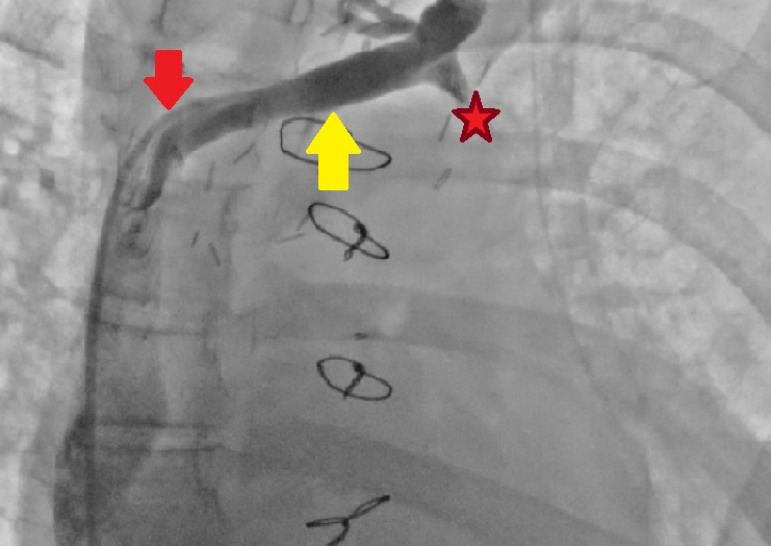
Postoperative cardiac catheterization. Yellow arrow=persistent left superior vena cava; red arrow=patent persistent left superior vena cava - right superior vena cava anastomosis; star=ligated stump of hemiazygos vein

Computed tomography angiography and cardiac catheterization prior to discharge showed that the PLSVC and the anastomosis were patent, and there was no sign of stenosis or thrombosis (Figures 2 and 3). Postoperative recovery of the patient was uneventful, and the patient was discharged with warfarin anticoagulation on the postoperative 5th day without any complications.

After one month of discharge, the patient showed no symptoms of venous stasis. Currently, the patient is asymptomatic on the 6th postoperative month. Timeline of the case report has been presented in the Table 1.

**Table 1:** Timeline of the case report.

Day 1	Admission to the emergency department	
	Symptom	Deep cyanosis
		Deteriorated medical condition
	Diagnosis	Echocardiography
		· Tetralogy of Fallot with severe pulmonary stenosis
		Cardiac catheterization
		· No additional abnormality
Day 2	Cardiac operation	Complete repair of Tetralogy of Fallot
		End-to-side anastomosis of the persistent left SVC to right SVC
Day 6	Postoperative CT angiography and cardiac catheterization	Anastomosis was patent
		No sign of stenosis or thrombosis
Day 7	Discharge	Discharged with warfarin anticoagulation
1^st^ and 6^th^ months after surgery		The patient is asymptomatic and shows no signs of venous stasis

CT=computed tomography; SVC=superior vena cava

## DISCUSSION

Systemic venous system abnormalities are generally benign anatomical structures that do not cause hemodynamic disturbances. However, sometimes these abnormalities can cause serious hemodynamic consequences on their own or in conjunction with additional congenital pathologies and may need to be corrected. Detection of these abnormalities prior to congenital heart surgery may provide the application of various surgical modifications during the operation.

PLSVC drains into the coronary sinus in 90% of all cases, and in only 8% of cases, the PLSVC is directly connected to the left atrium. The association of this situation with tetralogy of Fallot is much rarer. There are limited number of case reports describing this association in the literature^[1]^.

In cases where PLSVC drains directly into the left atrium, multiple surgical options are available. Ligation of PLSVC^[1]^, intra-atrial baffle formation^[2]^, transection of PLSVC with left atrial tissue and implantation to the right atrium^[3]^, end-to-side anastomosis of PLSVC to the left pulmonary artery^[4]^, a graft interposition between PLSVC and the right atrium^[5]^, forming an intra-atrial conduit with an inverted left atrial appendage flap^[6]^, and performing end-to-side anastomosis to the right SVC under or over the aortic arch^[7]^ are the surgical techniques reported in the literature^[2-7]^. Complex congenital pathologies are additionally detected in almost all cases where PLSVC is attached to the left atrium^[1]^. Therefore, it is necessary to evaluate each anatomical structure individually and to decide the most appropriate surgical technique to perform according to this anatomical structure.

In our patient, we preferred to perform an end-to-side direct anastomosis of the PLSVC to the right SVC for the following reasons:^[1]^the accurate diagnosis of the patient was confirmed during the cross-clamping period^[2]^, and the possibility of performing the anastomosis after complete repair and during the rewarming period on the beating heart^[3]^. We had some concerns regarding the possible complications of intra-atrial baffle formation and extracardiac graft interposition techniques. In previous studies, it has been reported that the long-term patency of intra-atrial baffle formation is questionable due to the use of non-growing material and that it may become stenotic. Beside this, due to the lack of growth potential, extracardiac graft interposition is also not applicable for young children^[1,4]^. In addition, these grafts may be compressed between the sternum and the aorta.

In their cohort studies, van Son et al.^[6]^, Ugaki et al.^[8]^, and Cesnjevar et al.^[9]^ showed that anatomic end-to-side anastomosis of PLSVC to the right SVC, anterior to the aortic arch, is a safe, feasible, and reproducible technique. Kawada et al.^[3]^ and Reddy et al.^[5]^ performed the anastomosis by redirecting the PLSVC under the aortic arc. Since PLSVC runs through the mediastinum more posteriorly than the right SVC, they suggested a more natural connection between the two SVCs could be achieved. The authors state that while performing this procedure, one must be sure that there is sufficient space between the main pulmonary artery and the aorta. Otherwise, the PLSVC may be compressed between these two structures.

Since the ascending aorta of the patient was dilated due to aortopathy (ascending aorta: 2.8 cm; Z-score: +3.79), we anticipated that the redirection of the PLSVC between the patient’s aorta and the main pulmonary artery, which was augmented transannularly, would also cause compression. As defined in the literature, the vena cava was extensively dissected free, and proximal branches were ligated and separated^[2,6,10]^, thus, approximately 3.5-4 cm of the PLSVC length was obtained. The PLSVC was transected from the left atrium roof and redirected over the anterosuperior part of the ascending aorta. Anastomosis was comfortably performed to the medial wall of the right SVC. No kinking or tension was observed in the anastomosis.

The most significant disadvantage of this technique is the lack of knowledge concerning long-term vascular patency. In the literature, the longest follow-up period was 2.4 years^[9]^. Studies with a longer follow-up period and larger patient cohorts are required to prove the durability of PLSVC and patency of anastomosis.

## CONCLUSION

In conclusion, there are multiple surgical options for patients with PLSVC draining directly into the left atrium. All these surgical techniques have their own shortcomings. Considering the sample cases in the literature, we believe that end-to-side direct anastomosis of the PLSVC to the right SVC might be a safe and applicable technique for pediatric patients with PLSVC draining into the left atrium.
